# The Predictors of New-Onset Atrial Fibrillation in End-Stage Renal Disease Patients: A Multi-Center Retrospective Observational Study

**DOI:** 10.3390/jcm15082879

**Published:** 2026-04-10

**Authors:** Ahmed A. Zayed, Mohamad Bahij Moumneh, Bahy Abofrekha, Hadi Itani, Omar Khayat, Abdelrahman Abouelnas, Martin M. Amor

**Affiliations:** 1 Department of Medicine, Northwell Health Staten Island University Hospital, 475 Seaview Ave., New York, NY 10305, USA; mmoumneh@northwell.edu (M.B.M.); babofrekha@northwell.edu (B.A.); hitani@northwell.edu (H.I.); okhayat@northwell.edu (O.K.); aabouelnas@northwell.edu (A.A.); 2 Department of Cardiology, Northwell Health Staten Island University Hospital, 475 Seaview Ave., New York, NY 10305, USA

**Keywords:** atrial fibrillation, chronic kidney disease, dialysis, racial disparities, risk factors, end-stage renal disease

## Abstract

**Background/Objectives**: Atrial fibrillation (AF) is a significant and prevalent cardiovascular complication in end-stage renal disease (ESRD) patients, yet its specific predictors within this population are not well understood. This study aimed to identify the predictors of new-onset AF in ESRD patients undergoing dialysis. **Methods**: We conducted a retrospective multicenter cohort study within the Northwell Health System, analyzing data from 5326 ESRD patients on dialysis between 2017 and 2019. The mean age was 64.2 ± 13.1 years, with 48.3% females. Multivariable-adjusted logistic regression was used to identify predictors of new-onset AF, the primary outcome. Adjusted odds ratios (ORs) were calculated for potential risk factors. **Results**: Of the 5326 patients, 1564 (29.4%) developed new-onset AF. Significant predictors included increasing age (OR 1.038 per year, 95% CI 1.032–1.044, *p* < 0.0001), obesity (BMI > 30 vs. BMI 20–25: OR 1.208, 95% CI 1.026–1.422, *p* = 0.023), and coronary artery disease (OR 1.678, 95% CI 1.466–1.920, *p* < 0.0001). African American patients had lower odds of developing AF compared to White patients (OR 0.578, 95% CI 0.494–0.676, *p* < 0.0001). Hypertension, sex, diabetes, and tobacco use were not significantly associated with AF risk. **Conclusions**: Age, obesity, and coronary artery disease are significant predictors of new-onset AF in ESRD patients on dialysis. Notable racial disparities exist, with African American patients having a lower risk. These findings may inform targeted prevention strategies and guide future research into the mechanisms underlying AF in ESRD patients.

## 1. Introduction

Atrial fibrillation (AF) is the most common type of heart arrhythmia and significantly impacts public health, contributing to 183,321 deaths and being the primary cause of 26,535 deaths in the United States in 2019 alone, according to the Centers for Disease Control and Prevention (CDC) [1]. Globally, AF prevalence is rising, with the 2021 Global Burden of Disease (GBD) study estimating that over 52 million individuals were affected. Projections indicate that AF will affect an estimated 12 million individuals in the US by 2050 [2]. Concurrently, End-Stage Renal Disease (ESRD), the final stage of chronic kidney disease (CKD), represents a critical health crisis, affecting 37 million adults in the US and over 670 million worldwide according to the 2021 GBD study, ranking as one of the leading causes of death [3]. ESRD necessitates dialysis due to the complete failure of kidney function.

The relationship between CKD and AF has been well-documented in prior research, which suggests that shared risk factors contribute to the increased prevalence of AF among CKD patients [2,3]. Additionally, AF may exacerbate CKD progression through altered cardiac hemodynamics, further complicating patient outcomes [4]. Incident AF in patients with CKD increases the rate of progression to ESRD by 67%, highlighting the bidirectional interaction between AF and CKD [5]. Despite these insights, a critical gap remains in understanding the specific predictors and outcomes of new-onset AF in patients with ESRD [6].

The results of Shen et al. (2016), a nationwide population-based study, indicated an increased incidence of AF among dialysis ESRD patients, identifying comorbidities such as hypertension, diabetes mellitus, hyperlipidemia, coronary artery disease, heart failure, valvular heart disease, and chronic obstructive pulmonary disease as significant risk factors [7]. However, this study did not focus on long-term predictors of new-onset AF in this group. Similarly, Hawerroth et al. (2023) highlighted the prevalence of AF in ESRD patients undergoing hemodialysis, yet these studies often lack a comprehensive analysis of the factors leading to new-onset AF in this population [8].

This study aims to fill this gap by providing comprehensive documentation of the predictors of new-onset AF in ESRD patients over two years within our U.S.-based hospital system. By analyzing data from multiple healthcare centers, this research seeks to compare findings and provide a robust understanding of AF risk and prognosis in the ESRD population. The goal is to guide strategies for preventing, diagnosing, and treating AF in ESRD patients more effectively.

## 2. Materials and Methods

### 2.1. Population and Sample

This multi-center retrospective observational cohort study was conducted within the Northwell Health System. The study population included patients diagnosed with ESRD who were undergoing dialysis. The initial data set consisted of 8734 patients identified between 1 January 2017 and 31 December 2019. Patients were stratified by AF status, identifying 1984 cases with new-onset AF and 6750 controls without AF. New-onset AF was defined as the first documented diagnosis of AF during the study period in patients without a prior history of AF. After removing duplicates and Patients with missing or physiologically implausible BMI values (BMI < 10 kg/m^2^ or >80 kg/m^2^) and those with incomplete tobacco use data were excluded, resulting in a final analytical cohort of 5326 patients from an initial pool of 8734.

### 2.2. Data Collection

Data was extracted from electronic health records of the Northwell Health System. Variables collected included demographic information (age, sex, race, ethnicity), clinical characteristics (BMI, hypertension, diabetes, coronary artery disease [CAD]), and lifestyle factors (smoking status). New-onset AF was identified using ICD-10 diagnostic codes documented in the electronic health records of the Northwell Health System, while ESRD diagnosis and dialysis details were obtained from nephrology records.

### 2.3. Statistical Analysis

Descriptive statistics were used to summarize the characteristics of the study population. Continuous variables were expressed as means ± standard deviations, while categorical variables were presented as frequencies and percentages. Multivariable-adjusted logistic regression analysis was performed to identify predictors of new-onset AF. Logistic regression was chosen as the primary analytical method, as AF status was captured as a binary outcome (present vs. absent) during the fixed study period. Variables included in the adjusted analysis were age, BMI, CAD, sex, race, ethnicity, hypertension, diabetes, and smoking status. Odds ratios (ORs) with 95% confidence intervals (CIs) were calculated for each predictor.

## 3. Results

### 3.1. Study Population

The final analysis included 5326 ESRD patients, of which 1564 (29.4%) were identified as cases with new-onset AF, and 3762 (70.6%) were controls without AF. The mean age of the study population was 64.2 ± 13.1 years. The cohort consisted of 48.3% females and 51.7% males. Racial distribution included 41.2% White, 35.4% African American, 15.6% Hispanic, 5.5% Asian, and 2.3% other/multiracial individuals.

### 3.2. Baseline Characteristics by AF Status

Table 1 presents the distribution of key demographic and clinical factors across patients with and without new-onset AF.

### 3.3. Predictors of New-Onset AF

Multivariable-adjusted logistic regression analysis identified several independent predictors of new-onset AF in ESRD patients. The results, including odds ratios, 95% Wald confidence intervals, and *p*-values, are presented in Table 2.

As shown in Table 2, significant predictors of new-onset AF included age (OR = 1.039; 95% CI: 1.032–1.044; *p* < 0.0001) and CAD (OR = 1.678; 95% CI: 1.466–1.920; *p* < 0.0001). For race categories (with White as the reference), the odds ratio for African American/Black was 0.578 (95% CI: 0.494–0.676; *p* < 0.0001), for Asian was 0.795 (95% CI: 0.629–1.005; *p* = 0.0552), and for Multiracial was 0.763 (95% CI: 0.598–0.974; *p* = 0.0301). BMI also showed a significant association with new-onset AF, with BMI > 30 compared to BMI 20–25 (reference category) showing an OR of 1.208 (95% CI: 1.026–1.422; *p* = 0.023). Other variables such as sex, ethnic status, diabetes mellitus, hypertension, and tobacco use did not show a statistically significant association with new-onset AF in this multivariable model.

## 4. Discussion

Atrial fibrillation is a prevalent and complex complication in patients with end-stage renal disease undergoing dialysis, with a multifactorial etiology involving structural, hemodynamic, inflammatory, and metabolic factors unique to the ESRD population. In our study, which analyzed over 5300 ESRD patients across multiple centers, we identified increasing age, obesity, and coronary artery disease as statistically significant independent predictors of new-onset AF, though the effect sizes were modest. Specifically, age showed a consistent association with new-onset AF (OR 1.038 per year; 95% CI: 1.032–1.044; *p* < 0.0001), representing a cumulative 46% increase in odds per decade. CAD demonstrated the largest effect size among the predictors examined (OR 1.678; 95% CI: 1.466–1.920; *p* < 0.0001). Obesity (BMI > 30 vs. BMI 20–25) was also a statistically significant predictor, though with a modest effect size (OR 1.208; 95% CI: 1.026–1.422; *p* = 0.023). Interestingly, hypertension, diabetes, sex, and tobacco use were not significantly associated with AF risk in this population, while African American race appeared to be protective, with a lower odds ratio (OR 0.578; 95% CI: 0.494–0.676; *p* < 0.0001) compared to White patients.

### 4.1. ESRD-Specific Pathophysiological Mechanisms

The pathophysiology of AF in ESRD patients differs substantially from that in the general population, with uremia-related mechanisms playing a dominant role. Uremic toxins, particularly indoxyl sulfate, have been shown to directly promote atrial arrhythmogenesis through multiple mechanisms [5,9,10,11,12]. Indoxyl sulfate causes calcium leak in the pulmonary veins, induces delayed afterdepolarizations, shortens left atrial action potential duration, and increases burst firing within the pulmonary veins—all electrophysiological changes that predispose to AF [5,11]. Animal studies have demonstrated that administration of AST-120, an absorbent of uremic toxins, decreases uremic toxin-induced AF, and clinical studies have shown that patients with higher serum indoxyl sulfate levels exhibit higher rates of AF recurrence after catheter ablation [9,10,12].

Beyond uremic toxins, ESRD patients experience chronic inflammation, oxidative stress, volume overload, and myocardial fibrosis—all of which contribute to atrial remodeling and AF substrate formation [5]. Volume overload, in particular, has been identified as an independent predictor of AF in ESRD patients. A prospective study using body composition monitors found that fluid overload (defined as overhydration/extracellular water ratio > 15%) was strongly associated with AF prevalence (OR 6.8, 95% CI 1.7–26.5, *p* = 0.006) and remained an independent predictor alongside age and coronary heart disease [13]. Left atrial stretch from volume overload promotes arrhythmogenesis through mechanical and electrical remodeling [5].

### 4.2. Traditional Risk Factors in the ESRD Context

The association between age and AF in our cohort is consistent with established mechanisms of age-related atrial fibrosis, electrical remodeling, and impaired conduction pathways [14,15,16]. This aligns with prior observations in both the general population and ESRD cohorts, reinforcing the need for early rhythm monitoring strategies in older dialysis patients. While obesity (BMI > 30) reached statistical significance as a predictor of new-onset AF in our model, the effect size was modest, and its clinical significance in the ESRD population should be interpreted cautiously. The BMI-AF relationship in dialysis patients is complicated by the “obesity paradox” and by the confounding effects of fluid status on body weight measurements. Nonetheless, the mechanistic role of adiposity in promoting atrial structural remodeling and inflammation is supported by prior studies demonstrating a positive correlation between epicardial fat tissue thickness and AF risk in hemodialysis patients. Epicardial fat tissue thickness, a direct marker of adiposity, has been positively correlated with AF risk in hemodialysis patients [17].

Coronary artery disease was the most potent predictor in our model. CAD-related ischemia, atrial strain, and myocardial fibrosis contribute significantly to AF pathogenesis in both dialysis and non-dialysis populations. Furthermore, elevated high-sensitivity cardiac troponin, a marker of cardiac injury, has been shown to predict cardiovascular outcomes in stable maintenance hemodialysis patients [1]. Studies have shown that elevated troponin T (>50 ng/L) and enlarged left atrial volume index (>30 mL/m^2^) are independently associated with the incidence of new-onset AF in patients with severe CKD [18].

Left atrial enlargement and diastolic dysfunction are more common in patients with CKD than without, and these structural changes are strongly associated with AF [1]. Echocardiographic studies in ESRD patients have demonstrated that those with AF have significantly larger left atrial volume index, longer atrial conduction delay, and reduced atrial function compared to those without AF [19]. In one study, ESRD patients with AF had a mean left atrial volume index of 29 ± 11 mL/m^2^ versus 23 ± 10 mL/m^2^ in those without AF (*p* = 0.001), and larger left atrial volume index was independently associated with AF on multivariable analysis [19].

### 4.3. Dialysis-Specific Risk Factors Not Captured

Interestingly, traditional risk factors such as hypertension and diabetes were not significantly associated with AF in our ESRD population. This finding may reflect a pathophysiologic override by volume overload, uremic cardiomyopathy, and dialysis-related hemodynamic fluctuations, which appear to exert a more dominant influence in ESRD than in the general population [5,13]. Moreover, studies in hemodialysis patients have linked intradialytic blood pressure variability and intradialytic hypotension to increased incident AF, emphasizing that the dynamics of blood pressure, rather than chronic hypertension per se, may be more critical in this population [5,20]. Nonetheless, the absence of significant associations for hypertension and diabetes in our model warrants careful interpretation, as both have been identified as significant predictors in prior CKD/AF registries, including the large population-based study by Shen et al. (2016) [7].

Our findings also align with the importance of dialysis-specific risk factors that were not captured in our dataset. Rapid ultrafiltration and high blood flow rates during hemodialysis have been shown to significantly increase the likelihood of incident AF [2]. A large US study of 15,414 hemodialysis patients found that ultrafiltration rate > 13 mL/h/kg was associated with 19% higher hazard of incident AF (adjusted HR 1.19, 95% CI 1.07–1.30), with risk beginning at rates of approximately 6 mL/h/kg [6]. These insights emphasize the importance of hemodynamic stability and individualized fluid removal protocols in mitigating AF risk. Additionally, AF occurs significantly more often during the dialysis procedure itself, suggesting that the acute hemodynamic and electrolyte changes during dialysis are important triggers [20].

The absence of association between smoking and AF in our study also deviates from findings in the general population, suggesting again that non-traditional ESRD-specific mechanisms may predominate. Reducing uremic toxin burden through adequate dialysis has also been associated with a lower risk of AF development [11].

### 4.4. Racial Disparities

The racial disparities observed, particularly the lower AF incidence in African American patients, mirror findings from prior studies [3,5]. While the mechanisms are complex and may involve genetic, anatomical, and autonomic differences, altered extracellular matrix homeostasis in response to hypertension has been proposed as a potential factor contributing to the lower AF incidence in African Americans despite a higher prevalence of conventional risk factors [9]. Studies have shown that African American patients have smaller left atrial size and different patterns of atrial remodeling compared to White patients, which may contribute to the lower AF prevalence [3,5]. However, it is important to note that ascertainment bias may also play a role, as AF detection may be lower in certain racial groups due to differences in healthcare access and screening practices [16].

### 4.5. Strengths and Limitations

A key strength of this study is its large, multicenter design, which enhances the generalizability of our findings within similar healthcare systems. However, this study has several important limitations. First, its retrospective observational design, while providing real-world data from a large cohort, limits the ability to establish causality between predictors and new-onset AF. Second, reliance on electronic health records means that the accuracy of diagnoses and the completeness of data are dependent on clinical documentation, potentially leading to misclassification bias for certain variables.

Third, and most critically, the absence of dialysis-specific variables represents a major limitation that substantially reduces the clinical utility of our prediction model for ESRD patients. Our dataset did not include critical dialysis-specific predictors that the literature identifies as major AF risk factors, including ultrafiltration rate, intradialytic hypotension and blood pressure variability, fluid overload status (as measured by body composition monitoring), electrolyte fluctuations, and dialysis adequacy measures [2,5,6,7,13,20]. These dialysis-related hemodynamic and metabolic factors exert a dominant influence on AF risk in ESRD patients and may explain why traditional cardiovascular risk factors like hypertension and diabetes were not significant predictors in our model. Their omission should be considered when interpreting the magnitude and significance of the associations reported herein. Future studies incorporating these dialysis-specific parameters are essential to develop clinically useful AF prediction models for ESRD patients.

Fourth, we did not have data on left atrial size, heart failure status, valvular heart disease, or sleep apnea—all of which are established AF risk factors and are particularly relevant in the ESRD population [1,18,19]. Their omission may have led to an overestimation of the independent effects of the predictors retained in our model, particularly coronary artery disease, which shares pathophysiological pathways with heart failure and left atrial remodeling.

Fifth, the lower observed AF incidence in African American patients should be interpreted with significant caution. Beyond potential biological mechanisms, ascertainment bias likely plays a substantial role. Disparities in healthcare access, frequency of ECG monitoring, rates of cardiologist referral, and differential utilization of ambulatory cardiac monitoring devices may all contribute to lower AF detection in minority populations. Furthermore, socioeconomic barriers to subspecialty care and differences in the clinical threshold for pursuing AF diagnosis may further confound these observations. The observed racial differences should therefore be considered hypothesis-generating rather than reflective of true biological differences in AF susceptibility, and prospective studies with standardized AF screening protocols across racial groups are needed to disentangle detection bias from genuine pathophysiological differences. Sixth, while we adjusted for numerous covariates, unmeasured confounders inherent to observational studies could still influence the observed associations. Seventh, the study was conducted within a single hospital system, which may limit the generalizability of findings to other populations or healthcare settings. Lastly, the definition of new-onset AF was based on diagnostic codes; this coding-based approach has been validated in prior studies, with positive predictive values for AF identification using ICD codes in administrative databases ranging from 70–96%, depending on the population and validation methodology. Individual-level ECG adjudication was not performed in this study, which we acknowledge as a limitation [2,5].

## 5. Clinical and Public Health Implications

These findings have significant clinical and public health implications. First, routine AF screening in ESRD patients should be prioritized, particularly for older, obese, and CAD-affected individuals. The KDIGO 2024 Clinical Practice Guideline recommends opportunistic pulse-based screening (e.g., when taking blood pressure), followed by a 12-lead electrocardiogram if an irregular pulse is identified [21]. Given the high AF burden in this population and the prevalence of asymptomatic AF, more intensive screening strategies should be considered, including continuous or periodic electrocardiographic monitoring during dialysis sessions, handheld ECG devices for home monitoring, or implantable cardiac monitors in high-risk patients [2,22,23]. The 2023 ACC/AHA/ACCP/HRS Guideline notes that the prevalence of AF among patients with renal failure on dialysis is high, including asymptomatic AF, supporting the need for systematic screening approaches [2].

Second, weight management programs and cardiovascular risk reduction strategies should be integrated into ESRD care to mitigate AF risk. Given that obesity (BMI > 30) confers a 21% increased risk of AF in our cohort, and that every 5-unit increment in BMI confers a 19–29% increased risk of incident AF in the general population, targeted weight reduction interventions may have substantial impact [4].

Third, blood pressure management strategies in hemodialysis patients should be revisited, with an emphasis on minimizing large intradialytic fluctuations rather than focusing solely on pre-dialysis blood pressure targets. Strategies such as individualized ultrafiltration rates, sodium profiling, and avoidance of excessive fluid removal may help stabilize hemodynamics and reduce AF triggers [5,7,20]. More research is needed to determine optimal blood pressure targets and ultrafiltration strategies for AF prevention in dialysis patients.

### 5.1. Anticoagulation Considerations

An important clinical consideration not addressed by our study design is the management of anticoagulation in ESRD patients with AF. The 2023 ACC/AHA/ACCP/HRS Guideline provides specific recommendations for anticoagulation in this population [2]. For patients with AF at elevated risk for stroke and end-stage CKD (CrCl 15 mL/min) or on dialysis, it might be reasonable to prescribe warfarin (INR 2.0–3.0) or an evidence-based dose of apixaban for oral anticoagulation to reduce the risk of stroke (Class 2b recommendation, Level of Evidence B-NR) [2]. However, conflicting data exist as to whether AF is a risk factor for stroke in patients on dialysis, and the decision to anticoagulate must balance thromboembolic and bleeding risks through shared decision-making [2].

Recent evidence suggests that apixaban may have a more favorable safety profile than warfarin in dialysis patients. A network meta-analysis found that warfarin, dabigatran, and rivaroxaban were associated with significantly elevated major bleeding risk compared to apixaban formulations [24]. The AXADIA-AFNET 8 trial, a randomized controlled trial comparing apixaban 2.5 mg twice daily with warfarin in patients with AF on hemodialysis, found no significant differences in safety or efficacy outcomes, though both groups experienced high rates of cardiovascular events [25]. The 2023 ACC/AHA/ACCP/HRS Guidelines for the Diagnosis and Management of AF have recommended that apixaban 2.5 mg twice daily is the appropriate dose for patients with ESRD on hemodialysis [2]. Left atrial appendage occlusion may be an alternative strategy for stroke prevention in dialysis patients who cannot tolerate anticoagulation, though prospective data are limited [2,26].

### 5.2. Policy and Health Equity Implications

From a policy standpoint, racial disparities in AF risk highlight the importance of personalized medicine approaches in nephrology and cardiology. Efforts should be made to ensure equitable access to AF screening and treatment across racial and ethnic groups. However, clinicians should also be aware that ascertainment bias may contribute to lower AF detection rates in certain populations, and proactive screening strategies may be needed to ensure equitable diagnosis [16]. Further studies are needed to assess whether genetic screening could provide additional insights into AF susceptibility in diverse populations.

## 6. Future Research Directions

Future studies should prioritize investigation of the hemodynamic and biochemical mechanisms linking ESRD to AF, particularly the role of dialysis-specific factors. Prospective studies incorporating ultrafiltration rate, intradialytic blood pressure variability, fluid overload status (measured by body composition monitoring), electrolyte fluctuations (particularly calcium, potassium, and magnesium), and dialysis adequacy measures are essential. Additionally, studies should evaluate the temporal relationship between dialysis sessions and AF onset, as evidence suggests AF occurs more frequently during and immediately after dialysis [20,22].

Randomized controlled trials assessing the effectiveness of AF screening strategies in ESRD patients are needed. Comparative effectiveness studies of different screening modalities—including opportunistic pulse checks, handheld ECG devices, continuous monitoring during dialysis, and implantable cardiac monitors—would help determine the optimal approach for this population [2,21,23].

Intervention trials testing strategies to reduce AF incidence in ESRD patients should be conducted, including trials of individualized ultrafiltration protocols, blood pressure management strategies to minimize intradialytic variability, uremic toxin reduction strategies (such as AST-120), and weight management programs.

Recent evidence has also highlighted the importance of understanding AF determinants in the context of spontaneous cardioversion. A 2025 study by Mariani et al. (2025) demonstrated that determinants of spontaneous conversion to sinus rhythm in paroxysmal AF overlap substantially with the inverse of the predictors identified in our ESRD cohort [27]. This is particularly relevant to ESRD patients, who may experience transient AF episodes triggered by dialysis-related hemodynamic and electrolyte shifts. The propensity for spontaneous cardioversion may differ in ESRD patients compared to the general population due to the reversible nature of some AF triggers (e.g., acute volume overload, electrolyte disturbances during dialysis). Future studies should distinguish between persistent AF and paroxysmal AF with spontaneous cardioversion in ESRD populations, as the predictors and clinical implications may differ substantially.

Finally, exploring the genetic and environmental determinants of racial disparities in AF risk may help refine personalized approaches to cardiovascular care in dialysis patients. Understanding why African American patients have lower AF incidence despite higher burden of traditional risk factors could provide insights into protective mechanisms and novel therapeutic targets.

## 7. Conclusions

Our findings underscore the unique landscape of AF risk in ESRD patients, where traditional cardiovascular risk factors may be superseded by ESRD-specific factors. Increasing age, obesity, and coronary artery disease were identified as statistically significant, though modestly sized, independent predictors of new-onset AF in this vulnerable population. The observed lack of association with hypertension, diabetes, and tobacco use, coupled with racial disparities, suggests a dominant role for uremia- and dialysis-related pathophysiological mechanisms, including uremic toxins (particularly indoxyl sulfate), chronic inflammation, volume overload, myocardial fibrosis, and dialysis-related hemodynamic fluctuations.

The absence of dialysis-specific variables in our analysis represents a critical limitation and highlights the need for future research incorporating ultrafiltration rates, intradialytic blood pressure variability, fluid status, electrolyte dynamics, and dialysis adequacy measures. Our findings should therefore be considered hypothesis-generating, and the reported associations require validation in prospective studies that capture the full spectrum of ESRD-specific risk factors before they can inform clinical prediction models. The integration of advanced cardiac biomarkers (such as troponin), echocardiographic measures (particularly left atrial size), body composition monitoring for fluid status assessment, and hemodynamic monitoring into routine care may significantly enhance AF prediction and prevention efforts, leading to more tailored and effective management strategies for AF in ESRD patients.

## Figures and Tables

**Table 1:** Baseline Characteristics of Study Population by AF Status.

Factor	No AF	AF Present
CAD		
No	2771 (74.93%)	927 (25.07%)
Yes	991 (60.87%)	637 (39.13%)
Weight		
Underweight	122 (73.05%)	45 (26.95%)
Healthy weight	1216 (70.25%)	515 (29.75%)
Overweight	1237 (71.71%)	488 (28.29%)
Obese	1187 (69.70%)	516 (30.30%)
Race		
White	1302 (61.79%)	805 (38.21%)
African American	1498 (80.15%)	371 (19.85%)
Other/Multiracial	602 (72.36%)	230 (27.64%)
HTN		
No	1330 (71.54%)	529 (28.46%)
Yes	2433 (70.15%)	1035 (29.85%)
Sex		
Female	1390 (70.77%)	574 (29.23%)
Male	2372 (70.55%)	990 (29.45%)
Diabetes		
No	1618 (69.83%)	699 (30.17%)
Yes	2144 (71.25%)	865 (28.75%)
Tobacco Use		
No	2711 (70.89%)	1113 (29.11%)
Yes	1051 (69.97%)	451 (30.03%)
Total	3762	1564

**Table 2:** Logistic Regression Analysis for Predictors of New-Onset AF.

Effect	Point Estimate	95% Wald Confidence Limits	*p*-Value
Age	1.039	1.032–1.045	*p* < 0.0001
Sex (Female vs. Male)	0.964	0.844–1.101	*p* = 0.5896
Race Category (African Amer/Black vs. White)	0.578	0.494–0.676	*p* < 0.0001
Race Category (Asian vs. White)	0.795	0.629–1.005	*p* = 0.0552
Race Category (Multiracial vs. White)	0.763	0.598–0.974	*p* = 0.0301
Ethnic Status (Hispanic/Latino vs. Not Hispanic or Latino)	1.058	0.802–1.396	*p* = 0.6894
BMI (Obese “BMI > 30” vs. Healthy weight “BMI 20–25”)	1.208	1.026–1.422	*p* = 0.023
BMI (Overweight “BMI 25–30” vs. Healthy weight)	0.955	0.816–1.118	*p* = 0.5675
BMI (Underweight “BMI < 20” vs. Healthy weight)	0.879	0.600–1.288	*p* = 0.5095
DM (Yes vs. No)	0.963	0.843–1.100	*p* = 0.5747
HTN (Yes vs. No)	1.12	0.990–1.280	*p* = 0.06
CAD (Yes vs. No)	1.678	1.466–1.920	*p* < 0.001
Tobacco Use (Yes vs. No)	0.888	0.771–1.022	*p* = 0.0981

## Data Availability

The original contributions presented in this study are included in the article. Further inquiries can be directed at the corresponding author.
